# Regular physical activity modulates perceived visual speed when running in treadmill-mediated virtual environments

**DOI:** 10.1371/journal.pone.0219017

**Published:** 2019-06-26

**Authors:** Martina Caramenti, Claudio L. Lafortuna, Elena Mugellini, Omar Abou Khaled, Jean-Pierre Bresciani, Amandine Dubois

**Affiliations:** 1 Department of Neuroscience and Movement Science, University of Fribourg, Fribourg, Switzerland; 2 Laboratorio di Biomeccanica “Franco Saibene”, Istituto di Bioimmagini e Fisiologia Molecolare, CNR, Segrate, Milano, Italy; 3 HumanTech Institute, University of Applied Sciences and Arts Western Switzerland, Fribourg, Switzerland; 4 Université Grenoble-Alpes, LPNC Grenoble, France; University of Minnesota, UNITED STATES

## Abstract

In virtual reality, visual speed is usually underestimated relative to locomotor speed. Here we investigated how physical activity and fitness affect perceived visual speed when running in a treadmill-mediated virtual environment. Thirty healthy participants (ten sedentary individuals, ten team sport players and ten expert runners) ran on a treadmill at two different speeds (8, 12km/h) in front of a moving virtual scene. Participants were asked to match the speed of the visual scene to their running speed (i.e. treadmill speed), indicating for each trial whether the scene was moving slower or faster than the treadmill. The speed of the visual scene was adjusted according to the participant’s response using a staircase until visual and running speeds were perceived as equivalent. More sedentary participants underestimated visual speed relative to their actual running speed. Specifically, visual speed had to exceed running speed to be perceived as equivalent. The underestimation of visual speed was speed-dependent, and it was significantly larger for sedentary participants than for team sports players and expert runners. The volume of physical activity per week was found to be the best predictor of visual speed perception for both running speeds, while the perceived effort constituted a good predictor only at 8km/h. Physical fitness, on the other hand turned out to be a poor predictor of visual speed perception. Therefore, in order to enhance users’ engagement and their adherence to physical activity programs, the development of “personalized” treadmill-mediated virtual environments should take into account users’ personal characteristics to provide the most natural and engaging feedback possible.

## 1. Introduction

Even though physical activity (PA) is associated with a decreased risk of chronic diseases and contributes to mental and overall well-being, current levels of inactivity are extremely high. This is partly due a low participation in PA during leisure time, and partly to the increase of sedentary activities during occupational time. As new technologies become more accessible, they should become useful tools to be integrated into PA programs in order to enhance the user’s engagement and to improve adherence.

Treadmills are widely used as a means to train cardio-vascular fitness indoor. Unfortunately, treadmills are often considered as boring as compared to outdoor activities [1]. Coupling treadmills and Virtual Reality (VR) into treadmill-mediated virtual environments could enhance the exercise experience by simulating outdoor environments. This might improve engagement, effort, and lead to adherence to longer PA programs. Studies have actually shown that the psychological benefits of exercise on energy and relaxation become more pronounced when PA is supported by this kind of technology, also resulting in a higher measured exertion (but not perceived exertion) and a greater enjoyment [2, 3]. Moreover, treadmill-mediated virtual environments could contribute to reduce the inconsistency between kinaesthetic/motor and visual information by providing appropriate visual information of movement.

The possibility to minimize sensory discrepancy and to improve engagement in PA is very attractive, but it greatly depends on the ability to personalize the settings of treadmill-mediated virtual environments. In particular, it is highly desirable that such environments be tailored to the user’s needs and preferences, notably taking into account perceptual aspects. This is important because in virtual and simulated environments, perceptual judgements can differ from those performed in the “real” world [4, 5]. Concerning more particularly locomotion, studies performed with walking [6–9] and running individuals [10] have shown that in virtual environments, visual speed is systematically underestimated relative to locomotor speed. With running individuals, larger visual underestimations were observed at higher running speeds [10], suggesting that visual underestimation is speed-dependent. This is interesting for the design of treadmills-mediated virtual environments because it means that the “preferred” and/or most natural gain between visual and locomotor speed changes with locomotor speed. But does this gain also change with the experience of the runner? More specifically, is the visual-locomotor gain affected by factors like the type of PA practiced, the amount of weekly practice, or the absolute level of fitness? This is an important question considering the large spectrum of users potentially working out on a treadmill. And this is a very legitimate question considering that those factors likely affect the preferred running speed of each user. In fact, spatial ability—i.e. the capacity to judge the relations between objects in space, shapes and sizes and to mentally manipulate objects or turn them over [11]—can be considered an integral part of some sports, especially those that require precise visuomotor coordination. Therefore, the cognitive abilities of athletes practicing different kinds of sports may differ based on the characteristics of the activity, training skills and expertise [12]. In line with this, some studies have shown that some functions related to visual processing, such as visualization, visual concentration, visual perception, reaction time and or visual search are more efficient in athletes than in non-athletes, and that the parameters of the visual system (i.e. dynamic visual acuity, visual reaction time, peripheral awareness, visual search ability) are better in professional athletes than in low-level athletes [13–15]. Also, some authors investigated whether individuals with experience of self-motion at high and variable speeds (i.e., athletes) were able to better calibrate idiothetic information (i.e., motion information based on self-motion cues and not on external cues such as landmarks) to update their internal representation of space, and whether they could use this information to guide locomotion more precisely than individuals without this experience (i.e., non-athletes) [16]. The results of these studies suggest that if a range of velocity is not used, at these velocities, people fail to appropriately calibrate and establish expected relationships between the differences sources of idiothetic information. Therefore, practice and training might help develop and improve this calibration process [17], influencing the processing of multi-sensory information at normal and fast velocities [16]. One can wonder if these differences also apply to speed perception in running, in which case one could observe differences in visual speed perception based on the running speeds and range of optical flows individuals are regularly exposed to.

Here we tried to determine whether PA affects perceived visual speed when running. More specifically, we assessed whether the type of activity regularly practiced can modulate perceived visual speed, and whether this modulation is the same at different running speeds [10]. We also assessed whether perceived visual speed is related to factors like the overall level of fitness of users, or the amount of PA practiced weekly.

## 2. Materials and methods

30 healthy participants (15 female, 15 male) with a mean age of 23.25 (±2.2 SD) participated in this study. They were naïve about the purpose of the study, had normal or corrected-to-normal vision and none had a history of cardiovascular disease. All participants gave their informed and written consent prior to their inclusion in the study that was performed in accordance with the ethical standards specified by the 1964 Declaration of Helsinki and approved by the Ethics Committee of the University of Fribourg.

The 30 participants were divided into 3 groups, each of them composed by 5 males and 5 females.

The groups were based on sport activity:

1. People that do not engage in sports (maximum 2 hours of light activity per week)–Sedentary
2. People engaging in sports that involve running (basketball, football, rugby, handball)–Team Sports
3. Expert runners—Runner

Prior to the running task, the participants filled out the short version of the International Physical Activity Questionnaire (IPAQ—French version) to get an overall estimate of their PA expressed in MET-min/week. METs, or metabolic equivalents, are used to describe the energy expenditure of an activity and are defined as the ratio of the energy rate expended during a specific activity and the energy expenditure at rest, which is 1MET. MET-min/week represent the volume of PA per week and are calculated as the sum of the metabolic equivalent levels of specific activities by the minutes of each activity per events per week.

**Fig 1:**
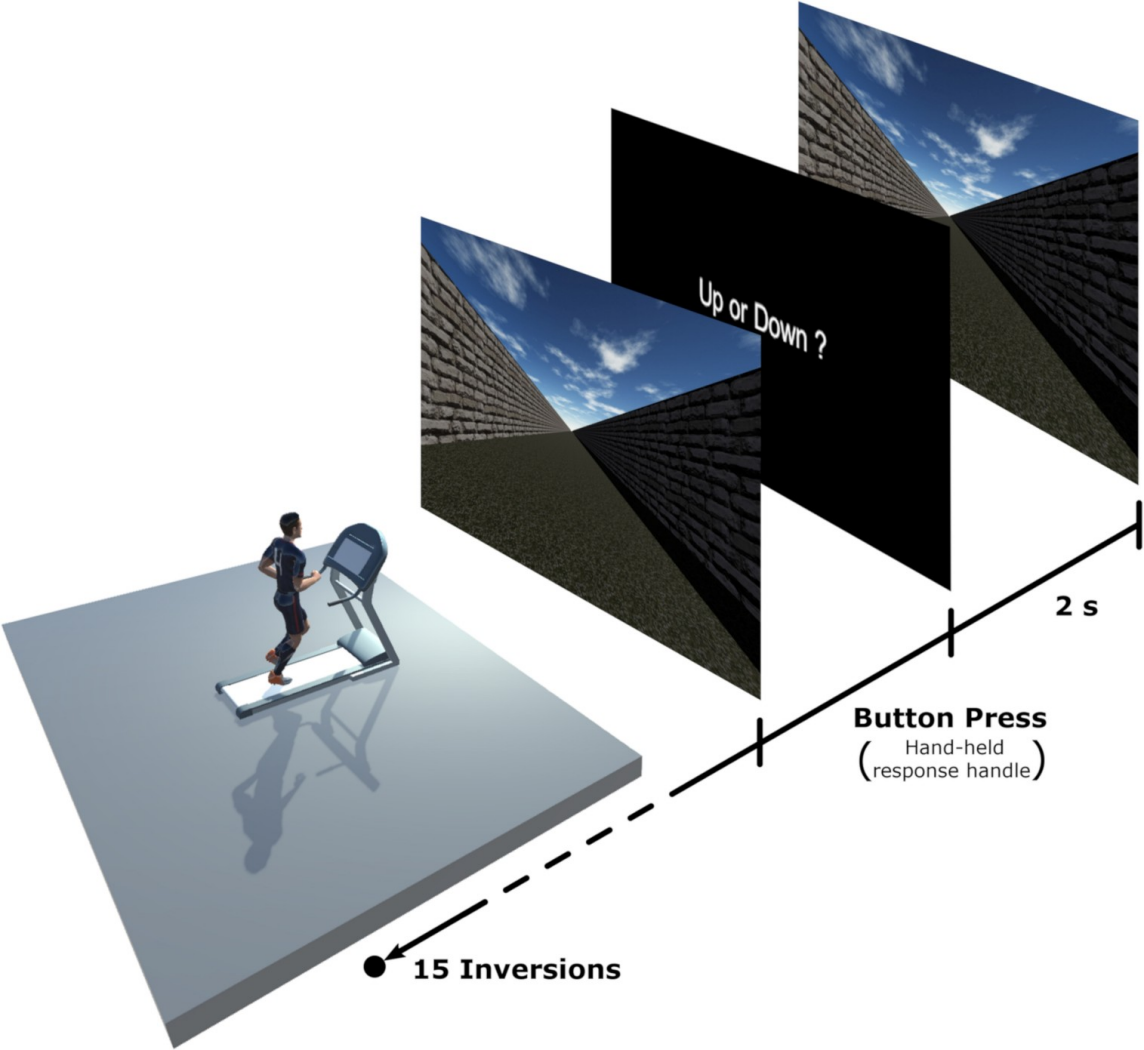
Schematic representation of the experimental set-up. Participants were running on the treadmill in front of a large projection screen (4.30x2.70m). For each trial, a moving visual scene representing an open-air hallway was briefly presented and participants were asked to indicate whether the scene was moving faster or slower than their actual running speed.

Participants ran on a HP Cosmos Mercury treadmill (Running surface: L:150cm, W:50cm). To simulate optical flow, a virtual environment created using Unity was viewed on a 4.30x2.70m screen positioned 2.56m in front of the treadmill, with an effective field of view of 80°. The scene was projected onto the screen using a Barco F50 WUXGA projector with a 1920x1200 pixels resolution. The VR scene for which judgements were made was a neutral open-air hallway presented at constant-velocity motion (Fig 1). Rich optical flow information was provided by the granular texture of the floor and the random pattern on the walls without giving any landmarks or usable spatial information. The room was darkened during the experiment, with the display screen being the only source of light. Participants ran holding light custom-made plastic cylinder (115x30mm, 15g), one for each hand. On the top of each cylinder was a response button that participants could effortlessly press while running to increase (left) or decrease (right) the speed of the visual scene. Participants’ responses were sent to the computer via Bluetooth.

While running, participants’ heart rate (HR) was constantly monitored (worn HR monitor Polar Team2 System, Polar Electro Oy—Finland).

The experiment consisted of two blocks. In each block, the participants ran at one of two different speeds (i.e., treadmill speeds): 8Km/h (2.23m/s) or 12Km/h (3.34m/s). The order of presentation of the two running speeds (i.e., blocks) was randomized and counterbalanced between participants. Each block was divided into 1-minute bouts, so as to alternate between slots of 1-minute run and 1-minute rest. While running, the participants were presented with a visual scene and instructed to estimate if the visual scene was slower or faster than the actual running speed. For each running speed, four consecutive tests were proposed in a random order using a one up one down staircase method [18, 19], starting two times with a visual speed higher (+4Km/h (+1.11m/s)) and two times with a visual speed lower (-4Km/h (-1.11m/s)) than the actual treadmill speed. The speed of the scenes was adjusted according to the one up one down staircase method, defining an increase/decrease of the visual speed of 0.5Km/h (0.14m/s) until the first inversion of the response, followed by steps of 0.3Km/h (0.08m/s). This method allowed us to determine the Point of Subjective Equality (PSE), which is a perceptual threshold indicating the visual speed that is perceived as “matching” the actual treadmill speed. For each of the actual running speeds, this threshold corresponds to the speed of the visual scene (optical flow) that is perceived as matching the actual running speed. Prior to the task, the participants spent a few minutes familiarising themselves with treadmill running at different speeds. They were then familiarized with the experimental setting and task using a training program, which presented the visual scene at different speeds. Experimental trials were initiated when the participant felt comfortable running at different speeds.

The single trials of each test started with the participant running at constant speed. Participants were instructed to gaze toward the end of the virtual hallway. The visual scene was presented for 2 seconds before the participants were challenged with a black screen presenting the question “Up or down?”:

- “Up” if visual speed appeared slower than the treadmill, so they wanted to increase the speed of the virtual scene
- “Down” if the visual speed appeared faster than the treadmill, so they wanted to decrease the speed of the virtual scene.

For each trial, participants gave their response by pressing the corresponding switch held in the left (Up) or right (Down) hand while continuing their treadmill run.

Once the participant pressed the chosen button, the graphics returned from the black screen to the visual scene of the following trial (see Fig 1). Each 1-minute bout started at the optical flow speed at which the previous bout ended.

Participants’ responses were sent via Bluetooth to the computer, so as to directly integrate them into the staircase test. Each staircase ended when 15 inversions of the responses were reached. At the end of each staircase test, the participants were free to take a short pause before starting the next test.

At the end of each 1-minute bout, the participants estimated their perceived exertion using the Borg RPE Scale (6 to 20 scale), ranging from “no exertion at all” to “maximal exertion”.

During a second session, the participants performed a 6-minute running test (6m-RT), as a means to estimate the Maximum Aerobic Speed (MAS). This test was carried out on track, with the participants instructed to run the longest possible distance in 6 minutes. The MAS is the lowest speed that elicits the maximal oxygen consumption (VO_2_max) during an incremental test, and it is considered as an indicator of the aerobic capabilities of the participant.

For statistical analysis, means where compared using t-tests or ANOVAs when data was parametric, and their non-parametric equivalent when data was not parametric. Normality and homogeneity of variance were systematically assessed with the Shapiro-Wilk test and the Levene test, respectively.

## 3. Results

For each group (i.e., Sedentary, Team sports, Runner) and each treadmill speed (i.e., 8 and 12km/h), we first compared the perceived visual speed with the actual running speed–i.e., the speed of the treadmill–using one-sample t-tests (perceived visual speed data was normally distributed for all 6 data samples). This allowed us to determine whether visual speed was over- or underestimated relative to running speed. For all 6 tests, the alpha level was corrected for multiple comparisons using Holm correction. For the Sedentary group, visual speed was set significantly higher than the actual treadmill speed (i.e., visual underestimation) for both treadmill speeds (t(9) = 3.397, p = 0.0079 for a treadmill speed of 8km/h, and t(9) = 4.802, p = 0.0009 for a treadmill speed of 12km/h). For the Team sports group, visual speed was set significantly higher than the treadmill speed only for a treadmill speed of 12km/h (t(9) = 6.0075, p = 0.0002). For the Runner group, there was no significant difference between the set visual speed and the actual treadmill speed, even though there was a tendency for a treadmill speed of 12km/h (t(9) = 0.0185, non-significant after correction for multiple comparisons).

**Fig 2:**
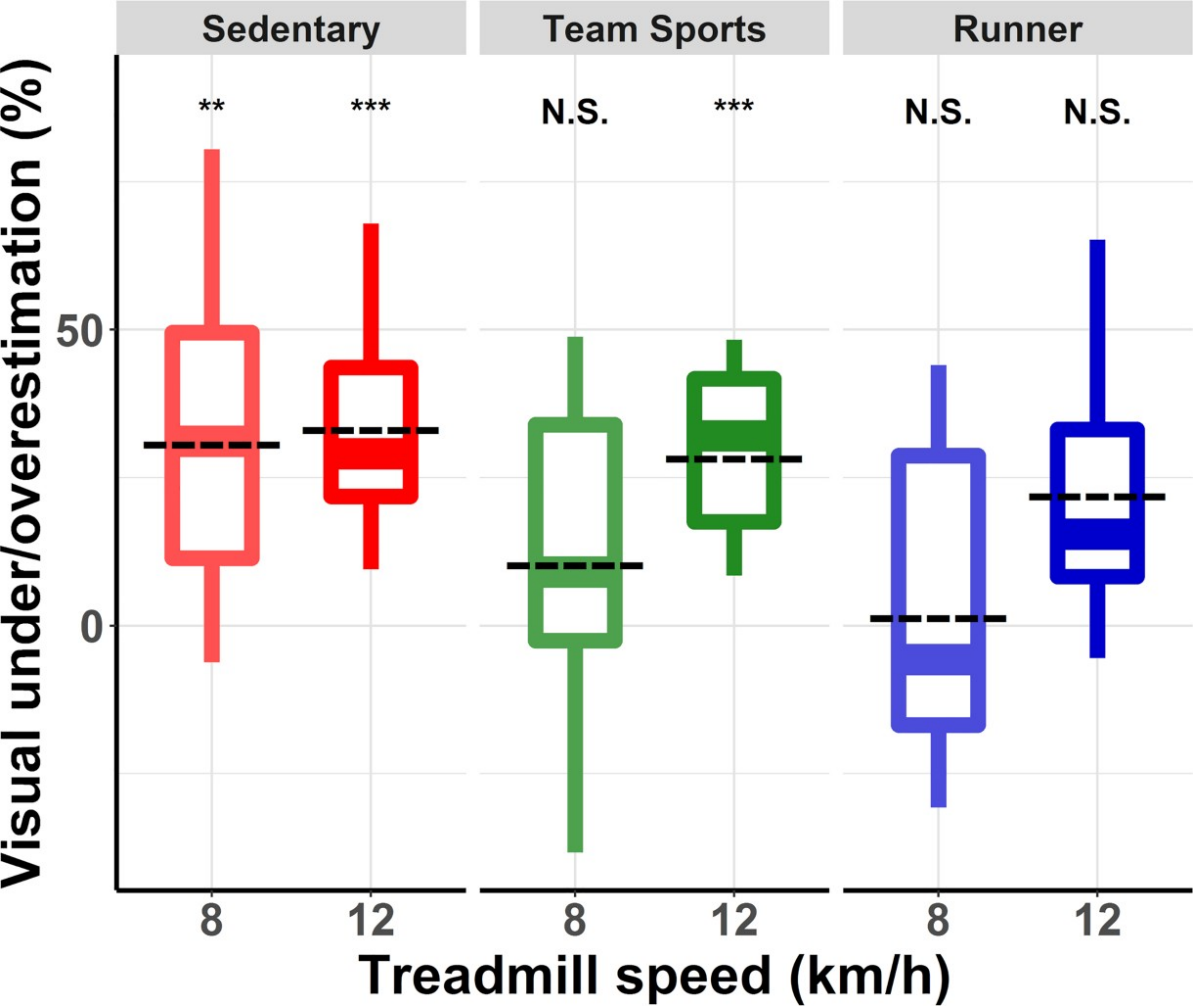
Percentage of underestimation (positive values) and overestimation (negative values) of visual speed relative to running speed. Estimations were computed using the equation: ln(perceived visual speed / actual treadmill speed) * 100. Each box summarizes the distribution of responses of the participants in each group and for each treadmill speed. The central line corresponds to the median, the box defines the inter-quartile range (IQR, between first and third quartile), and the whiskers correspond to ±1.5 IQR. Significant difference: N.S. = Not Significant, * = p<0.05, ** = p<0.01, *** = p<0.001.

We then tested whether PA and treadmill speed affected the amplitude of the visual underestimation of speed. For each group and each treadmill speed, we computed the percentage of visual under/overestimation using the equation: ln(*perceived visual speed/actual treadmill speed*)*100. This percentage indicated how much faster–for positive values–or slower–for negative values–the optical flow had to move relative to the treadmill speed for the two speeds to be perceived as equivalent. The logarithm (i.e., *ln*) was used because neither visual nor treadmill speed constituted an absolute reference value [20]. Fig 2 shows these percentages for the three groups at both treadmill speeds.

**Fig 3:**
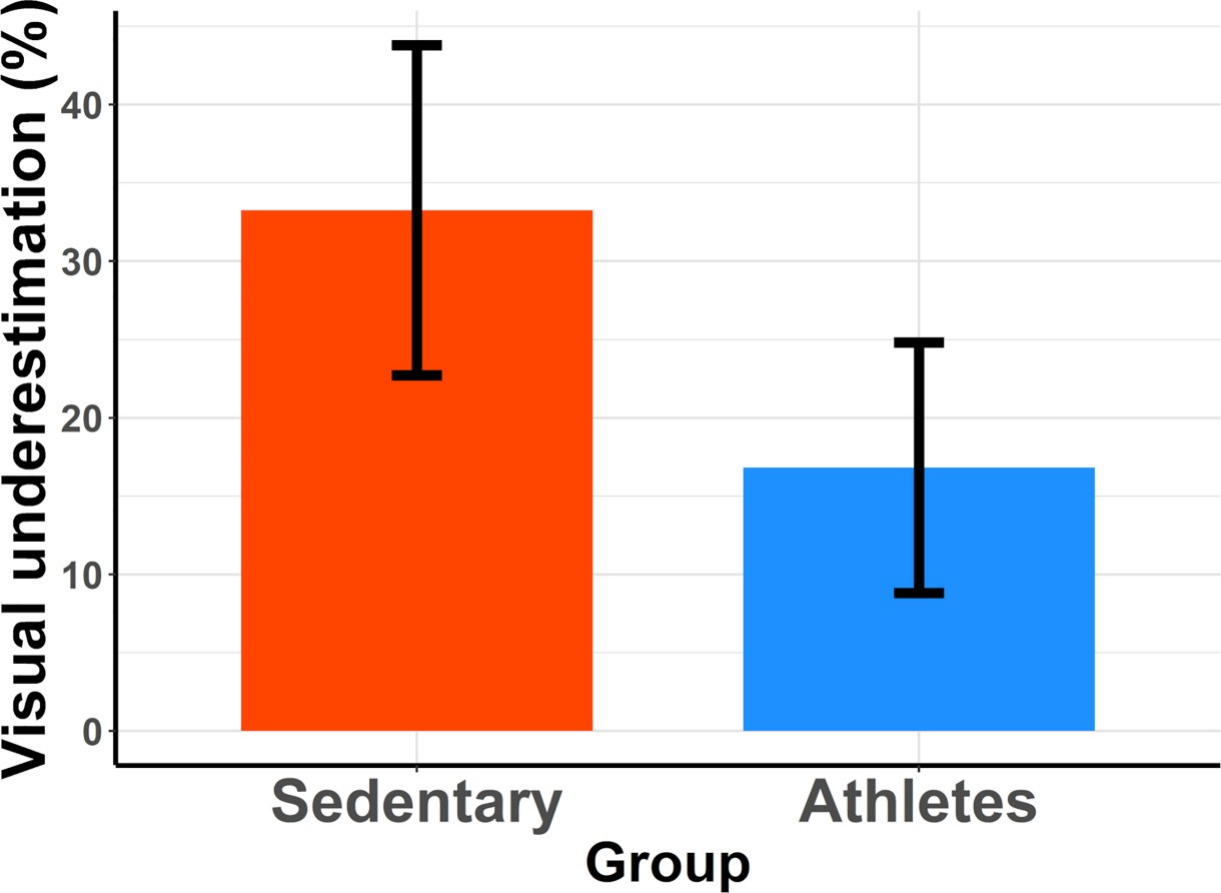
Mean visual underestimation in sedentary and physically active participants. Estimations were computed using the equation: ln(perceived visual speed / actual treadmill speed) * 100. The error bars represent the 95% confidence interval.

In a first step, we compared the mean underestimation values using a 3*2 (group[Sedentary, Team sports, Runner]*treadmill speed[8, 12]) mixed analysis of variance (ANOVA). The ANOVA revealed a main effect of treadmill speed [F(1,27) = 14.054, p = 0.0008, $\eta_{G}^{2}$ = 0.09]. Visual speed underestimation was larger for a treadmill speed of 12km/h (mean underestimation = 29.13%) than for a treadmill speed of 8km/h (mean underestimation = 15.46%). The main effect of the group failed to reach significance [F(2,27) = 2.331, p = 0.1164] but it nonetheless explained over 12% of the observed variance ($\eta_{G}^{2}$ = 0.12), and the underestimation varied from 33.25% for the Sedentary group to 20.64 and 12.99% for the Team sports and Runner group, respectively. There was no interaction between the main factors [F(2,27) = 2.408, p = 0.1091]. We then used orthogonal contrasts to compare the mean visual underestimation observed in the Sedentary group with that observed in the other two groups pooled together. In other words, we assessed whether we could identify a difference of underestimation between the group of sedentary people and the groups composed of physically active people. On average, visual underestimation in the Sedentary group was 16.44% larger than for physically active people (Team sports+Runner), as shown in Fig 3. This difference was significant (p = 0.016). A second contrast comparing visual underestimation between the Team sports and the Runner group indicated that there was no difference between those two groups of physically active people (p = 0.32). The mean difference of underestimation between these two groups was 7.65%.

For each group, we then compared the visual underestimation observed with a treadmill speed of 8km/h with that observed at 12km/h (see Table 1). This allowed us to assess how the actual running speed specifically affected visual underestimation in each group. We ran three targeted paired t-tests and adjusted the alpha for multiple comparisons using Holm correction. For the Sedentary group, the amplitude of visual underestimation did not vary between the two treadmill speeds [t(9) = -0.559, p = 0.589]. For both the Team sports and the Runner group, visual speed underestimation was larger at a treadmill speed of 12km/h than at a treadmill speed of 8km/h. This difference was significant for the Runner group [t(9) = -3.105, p = 0.012] but barely failed to reach significance for the Team sports group [t(9) = -2.392, p = 0.040 uncorrected, with an alpha adjusted to 0.025].

**Table 1:** Percentage of underestimation of visual speed relative to running speed. Estimations were computed using the equation: ln(perceived visual speed / actual treadmill speed) * 100. Values are means (SD).

	Running speed	
Group	8km/h	12km/h
Sedentary	32.0 (26.6)	34.5 (18.9)
Team Sports	11.6 (26.7)	29.6 (14.0)
Runners	2.7 (27.8)	23.3 (23.2)

In a second step, we assessed how activity and treadmill speed affected perceived effort and fitness-related variables (see Table 2).

**Table 2:** Fitness-related variables. Values are means (SD).

Groupe	Borg		HR		6m-RT	IPAQ
	8km/h	12km/h	8km/h	12km/h		
Sedentary	10.6 (2.0)	14.8 (1.5)	147.6 (23.6)	165.0 (20.3)	1258.5 (165.2)	1644.3 (983.1)
Team Sports	9.6 (1.8)	14.0 (1.6)	122.7 (10.4)	142.0 (10.6)	1506.0 (126.5)	5386.4 (2097.1)
Runners	9.0 (1.7)	12.6 (1.3)	117.3 (9.9)	137.5 (11.5)	1752.5 (152.8)	5833.8 (2333.2)

For both HR and Borg RPE (from now on just Borg), we used a 3*2 (group*treadmill speed) analysis of variance (ANOVA). For both parameters, the ANOVA revealed a main effect of sports activity (HR: F(2,27) = 10.46, p<0.001; Borg: F(2,27) = 4.449, p = 0.0214) and of treadmill speed (HR: F(1,27) = 292.998, p<0.001; Borg: F(1,27) = 180.380, p<0.001). For both variables, there was no interaction between the main factors. We then used orthogonal contrasts to identify any difference of HR and Borg between the group of sedentary people and the groups composed of physically active people. This difference was significant for both HR (p<0.001) and Borg (p = 0.003), while it failed to reach significance between the two active groups (HR: p = 0.317, Borg: p = 0.07).

The results of the 6m-RT and of the IPAQ (MET-min/week) were then compared between the three activity groups. For the 6m-RT, a one-way ANOVA indicated a main effect of the group [F(2,27) = 23.08, p<0.001]. Orthogonal contrasts indicated a significant difference between the Sedentary group and the other two groups pooled together (p<0.001), as well as between the two active groups (p = 0.004). Regarding the MET-min/week, a Kruskal-Wallis rank sum test indicated a main effect of the group [chi-squared(2) = 36.479, p<0.001). Multiple comparisons performed using a Dunn’s test showed a significant difference between the Sedentary group and both the Team Sports (p<0.001) and the Runner group (p<0.001).

Finally, a 3*2 ANOVA (group*treadmill speed) indicated that test duration was affected by treadmill speed [F(1,27) = 9.96, p = 0.004] but not by sports activity (p = 0.585).

These results indicate that overall, physically active participants had a higher level of fitness and a lower level of perceived effort (i.e., effort perceived as less intense) than their sedentary counterpart. Therefore, we assessed to which extent the amplitude of underestimation of visual speed could be explained/predicted by the measured “indicators” of fitness, perceived exertion, and the weekly volume of PA. For each of the two treadmill speeds, we used a multiple regression model with four predictors, namely performance on the 6m-RT, HR during effort, results of the Borg scale, and MET-min/week. At 8km/h, the regression model explained 53% of the variability of visual speed perception (R^2^ = 0.53, adjusted R^2^ = 0.46, p<0.001). Only the results of the Borg scale and the MET-min/week significantly contributed to the model (standardized beta value: 0.48 for the Borg scale and -0.53 for the MET-min/week). At 12km/h, the regression model explained 50% of the variability of visual speed perception (R^2^ = 0.50, adjusted R^2^ = 0.43, p<0.01). At this running speed, only the MET-min/week significantly contributed to the model (standardized beta value: -0.73). In other words, the MET-min/week was the “best” predictor of visual speed perception, for both running speeds. The results of the Borg scale also constituted a good predictor of visual speed perception, but only at a running speed of 8km/h. As for HR and the performance on the 6m-RT, both turned out to be poor predictors of visual speed perception.

## 4. Discussion

Participants running on a treadmill were instructed to match the visual speed of a moving scene to their actual running speed–i.e. the treadmill speed. Sedentary participants systematically set the speed of the visual scene higher than the actual treadmill speed. In other words, they systematically underestimated visual speed relative to their running speed. This was also the case for team players at 12 km/h, but not for runners. Interestingly, the amplitude of visual underestimation was affected by the PA of the participants, and more physically active participants showed an underestimation of lesser amplitude. However, the effect of PA on visual underestimation was not directly related to the level of fitness of the participants, suggesting that visual underestimation might rather relate to the mapping between visual, motor and kinaesthetic information.

Previous studies with walking [6–9], and running participants [10], reported a general tendency to underestimate visual speed with respect to locomotor speed. Our results confirm this tendency, as well as the fact that with running individuals, visual speed underestimation is speed-dependent [10]. Specifically, visual-locomotor gain increased with increasing running speed, mean gain ranging from 1.22:1 at a running speed of 8km/h to 1.36:1 at a running speed of 12km/h. Such a speed-dependent modulation of the visual-locomotor gain was never observed with walking individuals [8, 21]. But most importantly, our results show for the first time that PA can modulate visual speed perception, at least with running individuals. On average, visual underestimation was over 16% larger for sedentary than for physically active participants. Specifically, sedentary participants exhibited significantly larger underestimation gain (1.43:1) than team-sports players (1.26:1) and runners (1.18:1). And whereas visual underestimation was significant at both running speeds (i.e., 8 and 12km/h) for sedentary participants, it was non-significant for runners and significant only at 12km/h for team players.

Previous studies have reported that the perception of gait speed, geographical slant and distance can be biased by the perceived locomotor effort [22–25]. In line with this, we thought that perceived effort, which varies with running speed, might affect perceived visual speed. Specifically, when matching visual and running speed, the underestimation of visual speed is relative to running speed. If perceived running speed is overestimated with respect to the actual running speed, visual speed is likely to be set higher, which should result in a larger visual underestimation relative to the actual running speed. If this is the case, an increased perceived effort might lead to an overestimation of running speed, and thereby to a larger underestimation of visual speed. Perceived effort being directly affected by the level of fitness at a given exercise intensity, a higher level of fitness should result in a lower perceived effort. Interestingly, we could not measure any direct relationship between the amplitude of visual underestimation and the cardiovascular fitness of our participants. In particular, participants’ performance on the 6m-RT and their mean HR while running on the treadmill turned out to be poor predictors of visual speed perception.

On the other hand, the overall volume of PA, as estimated by the MET-min/week, proved to be the best predictor of visual speed perception. A higher volume of MET-min/week indicates a higher amount of time spent weekly in moderate and vigorous PA, which includes sport-specific training sessions and competitions. Note that a high volume of MET-min/week can be achieved as well with high amounts of walking, an activity that includes also intensities that do not have a major impact on cardiovascular fitness, probably explaining the only moderate correlation between the overall volume of PA and the indicators of cardiovascular fitness (HR: rho = -0.46; 6m-RT: rho = 0.56). Our results show that the more time participants spent weekly in PA, the less they underestimated visual speed. Taken together, these results suggest that PA, including not only moderate and vigorous PA but also walking, modulates the perception of visual speed relative to locomotor speed, but that this modulation cannot directly be imputed to the level of physical fitness per se or to perceived effort, and therefore likely results from other underlying factors.

One “fitness-independent” factor likely affecting perceived visual speed relates to the integration of visual and kinesthetic information about self-motion. Specifically, several studies have shown how the perception of travelled distance and motion speed can be affected by the integration and the calibration of visual and kinesthetic/motor cues [26–29]. Because we are less often exposed to self-generated, locomotion-induced visual flows at faster pace, the processing of visual speed information in this higher speed range might be less well “calibrated”. In particular, the visual flow resulting from self-motion might be less “tightly” associated/mapped to the motor and kinesthetic motion information. In other words, regular exposure to visual flows at higher velocity might give rise to a better “calibration” of the mapping between motion-evoked visual information and the corresponding kinaesthetic and motor information in this higher range of velocities. Along that line, Bredin et al. [16] tested if people with experience of self-motion at high and variable speeds (athletes) were able to better calibrate and use self-motion information to update their internal representation or space when compared to people lacking this experience (non-athletes). They found that when asked to reach a previously seen target by walking blindfolded at different speeds, the performance of athletes and non-athletes was more accurate at normal / preferred speed. At fast speeds, athletes were accurate while non-athletes tended to undershoot the target (i.e., walk a shorter distance). At slow speeds, both athletes and sedentary people tended to overshoot the target. These results suggest that when not used to move at given velocities–or within a given range of velocities, people might less adequately associate sensory signals related to self-motion (i.e., optical flow, biomechanical information, vestibular information) to their actual moving speed. This could explain the sports activity-related difference in visual speed perception observed in our study. Specifically, expert runners are used to run at constant speed at a variety of speeds. On the other hand, team sports that involve running are characterized by running patterns that include acceleration, deceleration, changes of directions and running at low speeds to recover. These running patterns seldom include middle to long distance running at a steady pace. In line with this, one can expect expert runners to better associate sensory signals about self-motion than team sports players. At the extreme end of the range, one can expect sedentary individuals who are very seldom exposed to different running speeds to have a harder time to map motion-evoked visual information to the corresponding kinaesthetic and motor information. These differences in running patterns could explain the observed difference in speed perception between athletes and sedentary people, but also the fact that team players significantly underestimated visual speed at a running speed of 12km/h, whereas the expert runners did not.

Also, according to the “mapping” theory of calibration, human motor control is regulated by one’s perception of the surrounding environment relative to the own perception-action system [30, 31], with perception providing the necessary information for selecting and guiding actions [32]. Studies show that thanks to practice and feedback, people are able to recalibrate the system and adopt a new mapping in case of disturbances of perceptual information [33, 34]. Furthermore, it seems that one can use past experience performing a certain target action to improve the accuracy of future judgements in the same situation [35, 36]. This is fundamental for a safe navigation through the environment [37], together with the fact that being in motion can facilitate perception [38] and perception of affordances, i.e. the possibilities for action [39]. This possibility to use past experience for recalibration could also have influenced the better visual speed estimation of expert runners, especially as compared to the sedentary group.

Unfamiliarity with treadmill running [40] might also have played a role in the altered perception of running speed observed in this study. In particular, increased attentional demand [41], differences in balance and coordination [42], and fear to fall off the treadmill are factors that could have contributed to speed overestimation. Moreover, treadmill running in itself has been shown to influence speed perception, treadmill locomotion being usually perceived as faster than the corresponding overground speed [43–46]. Along a similar line, the differences in running kinetics and kinematics between overground and treadmill running have been shown to influence the capacity to discriminate running speed [47–50]. These differences include longer periods of support on the stance leg, smaller vertical velocity of the centre of mass of the body, shorter stride length and higher stride rate for treadmill running [47, 51, 52]. Durgin et al. [21] have shown that for walking, the higher step frequency on treadmills could lead to biases in the perceived speed. This is likely because step frequency usually dominates in the estimation of walking speed [53–56]. In summary, the alterations in stride length and stride rate reported for treadmill running could also have contributed to the alteration of perceived locomotor speed.

## 5. Conclusion

PA practice is fundamental at all ages for health and disease prevention. With the recent advances in technology, treadmill-mediated virtual environments may become a valuable tool to improve engagement and adherence to PA programs. In particular, coupling virtual environments to treadmills would provide a more stimulating and enjoyable experience during indoors PA. In addition, it would contribute to reduce the discrepancy between kinaesthetic/efferent and visual information during treadmill locomotion. However, this requires a better understanding of the different physiological and perceptual mechanisms related to the use of VR in the PA practice. Our results suggest that when designing treadmill-mediated virtual environments, one should take into account the volume of PA of the users and the type of sports activity they practice in order to provide them with the most natural and engaging feedback possible.

## Supporting information

S1 Dataset Collection of recorded data. (CSV)
